# Octreotide infusion pump in patients with functional neuroendocrine tumors and refractory hormonal syndrome

**DOI:** 10.1530/EO-25-0016

**Published:** 2025-05-14

**Authors:** Kalyan Mansukhbhai Shekhda, Eleni Armeni, Dalvinder Mandair, Aspasia Manta, George Parker, Akanksha Sarma, Aimee Hayes, Martyn Caplin, Christos Toumpanakis

**Affiliations:** ^1^ Neuroendocrine Tumour Unit, ENETS *Centre of Excellence*, Royal Free Hospital NHS Foundation Trust, London, UK; ^2^ Endocrine Unit, 2nd Propaedeutic Department of Internal Medicine, Attikon University Hospital, Medical School, National & Kapodistrian University of Athens, Athens, Greece

**Keywords:** octreotide infusion pump, refractory hormonal syndrome, neuroendocrine tumors, carcinoid syndrome

## Abstract

**Objective:**

To evaluate clinical outcomes, safety and survival measures of octreotide infusion pump (OIP) therapy in patients with metastatic neuroendocrine tumors (NETs) and refractory hormonal syndrome.

**Design:**

A retrospective analysis was conducted using data from patients treated with OIP therapy at a single center.

**Methods:**

Data on demographics, disease characteristics, biochemical profiles and treatment outcomes were extracted from electronic patient records.

**Results:**

Eighteen patients with NETs and debilitating symptoms refractory to maximum approved doses of somatostatin analogs (SSTAs) were included. The cohort comprised 18 patients (12 males (67%) and six females (33%)) with a median age of 64.5 years (IQR: 49.5–71). The most common tumor site was midgut (72.2%), followed by pancreas (22.2%). Refractory carcinoid syndrome was the primary indication for initiation of OIP therapy in 15 patients and VIPoma in three. Most tumors were WHO grade 1 or 2 (89%), and liver metastases were prevalent (94% of patients). At presentation, the median 24-h urinary 5-hydroxyindoleacetic acid (5-HIAA) level was 421.5 μmoL/24 h (*n*: 8). The mean starting OIP dose was 1,632 ± 522 μg/24 h, escalating to 2,166.7 ± 464 μg/24 h in 66.57% of patients. Symptomatic improvement was observed in 72% of patients, significantly reducing flushing and diarrhea. Patients who did not respond well to OIP therapy had more disease burden and had received more treatment lines before being started on OIP therapy.

**Conclusion:**

OIP therapy is an effective treatment option for symptom control in patients with refractory NET-related hormonal syndrome. Randomized controlled trials are warranted to confirm these findings and assess long-term outcomes.

## Introduction

Neuroendocrine tumors (NETs) are rare neoplasms that arise from chromaffin cells within the diffuse neuroendocrine system throughout gastrointestinal, respiratory and other tissues (Sultana *et al.* 2023). These cells often secrete hormones such as amines, neuron-specific enolase, peptides, synaptophysin, metanephrines, non-metanephrines and chromogranin (Rindi *et al.* 2022). Based on their ability to secrete hormones, they are classified as either functional NETs (F-NETs) or non-functional NETs (NF-NETs) (Rindi *et al.* 2022). Based on their site of origin, they are further classified into pancreatic neuroendocrine tumors (Pan-NETs), gastrointestinal NETs (GI-NETs) and bronchial NETs. Neuroendocrine tumors (predominantly GI-NETs and bronchial NETs) can also cause a specific functional syndrome, carcinoid syndrome, which occurs mainly when liver metastases are present (>95% cases), but rarely with NETs that drain into the venous system circumventing the liver (bronchial, ovarian, testicular or pancreatic) (Ito *et al.* 2012, Oleinikov *et al.* 2019).

The clinical management of patients with F-NETs comprises control of disease progression and control of the hormone excess state (Ito *et al.* 2016). Patients with F-NETs often present with debilitating symptoms of variable degrees such as diarrhea, flushing, abdominal pain and bronchoconstriction, which can cause significant morbidity. These patients should be treated promptly, aiming to achieve maximum symptom control (Grozinsky-Glasberg *et al.* 2022). The first line of management for symptom control in patients with F-NETs is somatostatin analogs (SSTAs), often using short-acting SSTA (e.g., octreotide) for immediate symptom control in severe syndrome and then long-acting SSTAs (e.g., octreotide LAR or lanreotide autogel). Patients with mild to moderate symptoms may be initiated on long-acting SSTAs without the use of short-acting SSTAs. Other treatment options for symptomatic control include hepatic resection, cytoreductive surgery, hepatic transarterial embolization, transarterial chemoembolization (TACE) or transarterial radioembolization/selective internal radiotherapy (TARE/SIRT), peptide receptor radionuclide therapy (PRRT), everolimus combined with SSTAs, interferon-α and telotristat ethyl (Grozinsky-Glasberg *et al.* 2022).

Despite the efficacy of SSTAs as first-line agents, a proportion of patients never achieve successful control of their symptoms attributed to F-NETs despite their treatment with conventional doses of these drugs (Broder 2015, Ruszniewski *et al.* 2016). Moreover, refractory cases may develop over time, where standard approved doses of SSTAs cannot control the severity of symptoms attributed to hormone excess (Pavel *et al.* 2012, Baldelli *et al.* 2014, Pokuri *et al.* 2016). The management of refractory symptoms varies according to the disease burden and the patient’s response to treatment (Riechelmann *et al.* 2017). When symptoms are not controlled with administration of long-acting SSTAs, they are often combined with short-acting octreotide 100–500 μg every 6–8 h (Grozinsky-Glasberg *et al.* 2022). The data on managing patients with symptoms refractory to traditional treatments are limited to small prospective studies and case series (Kvols *et al.* 2012, Koffas *et al.* 2016, Molina-Cerrillo *et al.* 2016, Hörsch *et al.* 2017, Grozinsky-Glasberg *et al.* 2022).

High doses of octreotide LAR, greater than 30 mg/month, have been offered to patients with refractory symptoms secondary to gastrointestinal NETs (Broder 2015). In addition, octreotide intravenous infusion at rates of 50–150 μg/h has been proven to control carcinoid crisis in patients with NETs with minimal side effects (Seymour & Sawh 2013). However, no data are available in the literature examining the efficacy of a subcutaneous octreotide infusion pump (OIP) to treat patients with F-NETs and refractory hormonal syndrome. Our study aimed to assess the impact of a subcutaneous OIP in patients with refractory hormonal symptoms on clinical outcomes, safety and progression-free survival (PFS), and discuss the available literature on follow-up and treatment of these patients.

## Materials and methods

### Study design and population

A retrospective analysis was conducted on all patients with metastatic F-NETs and refractory hormonal symptoms managed with OIP at the Royal Free Hospital ENETS *Centre of Excellence*. These patients were identified from our central database, consisting of over 1,800 neuroendocrine neoplasms (NENs) patients under active surveillance and/or treatment, out of a more extensive database of more than 3,500 NENs patients seen between 2006 and 2023. Inclusion criteria were as follows: i) patients with metastatic disease secondary to NETs, ii) despite of receiving standard treatment for control of disease progression as well as treatment with the maximum labelled/approved dose of SSTAs, substantial symptomatic deterioration and poor quality of life at home and iii) debilitating and severe symptoms related to carcinoid syndrome (e.g., facial flushing, abdominal pain and/or diarrhea). All patients were investigated and/or treated for other causes of chronic diarrhea and abdominal discomfort, such as pancreatic exocrine insufficiency or small intestinal bacterial overgrowth, before diarrhea was solely attributed to F-NETs. All patients had courses of subcutaneous octreotide 200 μg twice or thrice daily, in addition to long-acting somatostatin analogs, with no significant improvement in symptoms before being started on a subcutaneous OIP.

For this study, we commenced treatment with a subcutaneous octreotide pump, which enabled continuous infusion of short-acting octreotide. The pump was provided as long as the patient continued to express debilitating symptoms. Symptoms were defined as debilitating if the patient reported any of the following: i) multiple daily episodes of facial flushing, ii) multiple episodes of diarrhea (Bristol stool chart type 6 or type 7), not controlled despite the use of maximum dose of loperamide and long-acting SSTAs with or without short-acting SSTAs. All patients were initiated on OIP therapy as inpatients, at a starting dose of 800 to 2,600 μg per 24 h via the BD BodyGuard™ T (Becton Dickinson Ltd, USA), a lightweight portable infusion pump was used with a cannula inserted subcutaneously either in the arm, thigh or abdomen, wherever it would be comfortable for the patient. The dose of octreotide infusion was determined based on severity of symptoms and tolerability of octreotide by the patients, generally starting with 50% more than their normal octreotide subcutaneous dose requirement. The dose of octreotide infusion was adjusted according to patients’ symptoms and response to the initial dose of octreotide while they were inpatients, and patients were discharged on a set dose of octreotide, which was continued in the outpatient setting. All patients were educated on the use of the pump and how to recognize signs indicative of pump operating failure. District nurse appointments were arranged to refill OIP with octreotide every 24 h at home. They were given contact details of our trained NET specialist nurse if they had persistent symptoms or if they needed any advice on the dose of OIP.

Since this study was a retrospective audit, ethical approval was not required under the UK Policy Framework for Health and Social Care Practice (registration number: RFHBU_89124/25).

### Data collection

Patients’ medical records were reviewed to collect data on demographics, disease characteristics, clinical and biochemical features, treatments received and outcomes. The tumor grade was determined based on the fifth edition of the World Health Organization (WHO) Classification of Endocrine and Neuroendocrine Tumors 2022 (Rindi *et al.* 2022).

Overall survival (OS) was defined as the time from OIP treatment initiation to the time of death from any cause or last date of contact for surviving patients. PFS was defined as the time from the start of OIP treatment to the time of disease progression. A symptomatic response (SR) was defined as an improvement of more than 50% in the patient’s symptoms. The maximum dose of octreotide in OIP was defined as the maximum dose to achieve SR.

### Statistical analysis

Continuous variables were expressed using mean values ±standard deviation (SD) or median values and interquartile range (IQR) (25th percentile–75th percentile) and range (minimum to maximum), while categorical variables were presented as absolute frequencies (*n*) and percentages (%). The normality assumption for each continuous variable was assessed using the Shapiro–Wilk test, combined with graphical methods. Progression was defined based on the review of cross-sectional imaging in the NET multidisciplinary board meeting (MDT) by NET-experienced radiologists as per Radiological Evaluation Criteria in Solid Tumors (RECIST) 1.1 (Morse *et al.* 2019). Survival analyses for OS and PFS were performed using the Kaplan–Meier estimation approach. All data analyses were conducted using SPSS software version 29.0.1.1, with the statistical significance level set at *P* < 0.05. All statistical tests were performed using a two-tailed approach.

## Results

A total of 18 patients with metastatic entero-pancreatic NET with complete information fulfilling the previously mentioned criteria were included in this study. At the time of data collection, four patients (22%) were still alive. Most patients were male (66.7%), with a median age at diagnosis of 64.5 (IQR: 49.5–71) years. Midgut was identified as the most common primary tumor site in 72.2% of patients, followed by the pancreas in 22.2%. Most patients were treated for refractory carcinoid syndrome (*n* = 15), while three were treated for VIPoma syndrome, also known as Verner-Morrison syndrome.

Histopathological analysis showed that 28% (*n* = 5) of the tumors were classified as grade 1 (G1), and 61% (*n* = 11) as G2, while data on grading were not available for two patients (11%). According to the biochemical data available at presentation (*n*: 8), the median 24 h urinary 5-hydroxyindoleacetic acid (5-HIAA) level was 421.5 (302.5–1,315) μmoL/24 h (RR: 0–47 μmoL/24 h). Out of three patients with VIPoma, only one patient had serum vasoactive intestinal peptide (VIP) levels available in our electronic patient record (EPR). His serum VIP levels were 250 pmol/L before initiation of OIP therapy and 384 pmol/L after initiation of OIP therapy (RR: 0–30 pmol/L). Regarding metastatic spread, most patients had liver metastases (*n* = 17), followed by mesenteric (*n* = 5), bone (*n* = 2), retroperitoneal (*n* = 2), pelvic (*n* = 2), para-aortic (*n* = 2) and mediastinal (*n* = 1) metastases.

The basic demographic and disease characteristics are summarized in Table 1.

**Table 1:** Demographics and disease characteristics.

	Median (IQR) or *n* (%)
Age at diagnosis (years)	64.5 (49.5–71)
Sex	
Male	12 (66.7)
Female	6 (33.3)
Primary tumor site	
Midgut	13 (72.2)
Pancreas	4 (22.2)
Unknown	1 (5.6)
Functional syndrome	
Carcinoid syndrome	15 (83.33%)
VIPoma	3 (16.66%)
Histology	
G1	5 (27.8)
G2	11 (61.1)
Unknown	2 (11.11%)
Urinary 5-HIAA (umoL/24 h) (*n*: 8)	421.5 (302.5–1,315)
Metastatic spread	
Liver metastases	17 (94.4)
Bone metastases	2 (11.1)
Retroperitoneal metastases	2 (11.1)
Mesenteric metastases	5 (27.8)
Pelvic metastases	2 (11.1)
Para-aortic metastases	2 (11.1)
Mediastinal metastases	1 (5.6)

5-HIAA, 5-hydroxyindoleacetic acid; IQR, interquartile range; VIPoma, vasoactive intestinal polypeptide tumors; G1, grade 1; G2, grade 2.

Prior to initiating OIP therapy, all patients had received previous treatments (Table 2). The majority had been administered SSTAs (*n* = 15), followed by PRRT (*n* = 13), surgical intervention (*n* = 11), systemic chemotherapy (*n* = 5) and molecular targeted therapies (*n* = 2). In addition, five patients with carcinoid syndrome had received telotristat ethyl for the symptomatic management of diarrhea. Patients had received a median of 3.5 (IQR: 1–8) prior lines of treatment. In patients who were on long-acting SSTAs before initiation of OIP therapy (*n*: 15), four patients (26.6%) were continued on long-acting SSTAs while being initiated on OIP therapy.

**Table 2:** Treatments before OIP initiation.

Treatment	*n* (%)
Long-acting ± short-acting SSTAs	15 (83.3)
PRRT	13 (72.2)
Surgical	11 (61.1)
Chemotherapy	5 (27.8)
Telotristat ethyl	5 (27.8)
Molecular targeted therapies (everolimus for one patient with metastatic midgut NET, sunitinib for one patient with pancreatic NET)	2 (11.1)

OIP, octreotide infusion pump; SSTAs, somatostatin analogs; PRRT, peptide receptor radionuclide therapy, NET, neuroendocrine tumor.

Major symptoms before SSTAs treatment and during SSTAs treatment but before OIP start are described in Table 3. The most common symptoms reported were flushing and diarrhea in 50 and 72% of patients while on SSTA treatment, respectively.

**Table 3:** Major symptoms before SSTA treatment and during SSTA treatment but before OIP start.

Symptoms before SSTAs	*n* (%)	Symptoms on SSTAs	*n* (%)
Flushing	11 (61.1)	Flushing	9 (50)
Diarrhea	10 (55.6)	Diarrhea	13 (72.2)
Shortness of breath	1 (5.6)	Shortness of breath	2 (11.1)
Syncope	1 (5.6)	Syncope	1 (5.6)
Nausea	1 (5.6)	Palpitations	2 (11.1)
		Carcinoid crisis	1 (5.6)

**Table 4:** Comparison of effect of OIP therapy between carcinoid syndrome and VIPoma.

	Carcinoid syndrome	VIPoma
Patients	15	3
Mean starting dose of octreotide in OIP therapy (in micrograms/24 h)	1,566.7 ± 456.2	2,466.7 ± 611.0
Mean maximum dose of octreotide in OIP therapy (in micrograms/24 h)	2,053.3 ± 443.8	2,600 ± 692.8
Symptoms improvement on OIP therapy (*n*: number of patients)	10 (66.66%)	3 (100%)
Side effects	1 (6.6%)	0 (0%)

OIP, octreotide infusion pump; VIPoma, vasoactive intestinal polypeptide syndrome.

**Table 5:** Patient demographics based on response to OIP.

	Patients who responded well to OIP	Patients who didn’t respond well to OIP
	Median (IQR) or *n* (%)	
Age (years)	74 (63–75)	77 (60.5–79.5)
Total number of patients	13 (72%)	5 (28%)
Sex		
Male	8 (62%)	4 (80%)
Female	5 (38%)	1 (20%)
Grading of tumor		
G1	0	2 (40%)
G2	10 (77%)	1 (20%)
Unknown	3 (23%)	2 (40%)
Disease burden		
Mesenteric mass	1 (8%)	0
Liver metastasis	7 (54%)	2 (40%)
Two sites of metastases	5 (38%)	0
Three or more sites of distal metastases	0	3 (60%)
Previous lines of treatment		
1	4 (31%)	0
2	2 (15%)	2 (40%)
3	1 (8%)	0
4	2 (15%)	2 (40%)
5 or more	4 (31%)	1 (20%)

OIP therapy was initiated typically around 47 months after diagnosis (median, range: 3–269 months). The mean starting dose of OIP was 1,632 ± 522 μg/24 h, which was increased to a mean maximum dose of 2,166.7 ± 464 μg/24 h, as 66.57% of patients (*n* = 12) required a dose escalation for better symptom management.

We also compared the effect of OIP on symptom control between carcinoid syndrome and metastatic VIPoma. The mean starting dose of OIP was higher in patients with metastatic VIPoma (2,466.7 ± 611.0 μg/24 h) compared to patients with carcinoid syndrome (1,566.7 ± 456.2 μg/24 h). All patients with metastatic VIPoma had SR after starting OIP therapy, whereas ten patients (66.66%) with metastatic midgut NET responded to OIP therapy (Table 4).

**Figure 1:**
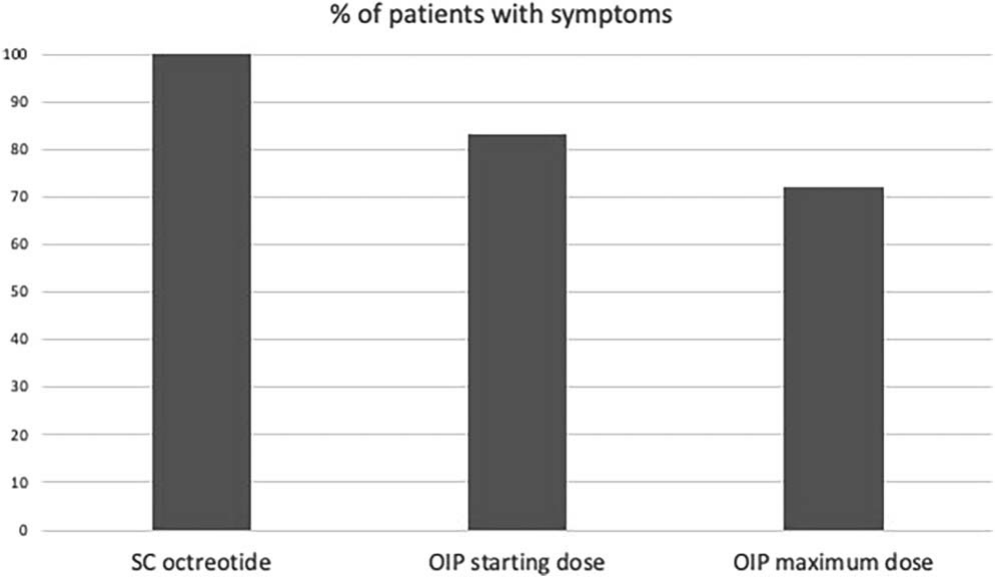
Percentage of patients exhibiting symptoms with each treatment category. SC, subcutaneous; OIP, octreotide infusion pump.

Although symptoms persisted in most patients, less percentage of patients had symptoms with the maximum OIP dose compared to the starting OIP dose and previous SSTA treatment (72.2 vs 83.3% vs 100%, respectively) (Fig. 1). Most patients reported a SR to OIP therapy (*n* = 13, 72%). Notably, out of 13 patients who had diarrhea before starting OIP therapy, ten patients (77%) reported SR. Out of nine patients with flushing, seven patients (78%) reported SR to OIP therapy.

Regarding the population who responded well to OIP, the majority of patients (*n*: 10, 77%) had grade 2 NET, whereas two (40%) and one (20%) patients had grade 1 and grade 2 NET in the cohort who did not respond to OIP, respectively. None of the patients had three or more sites of metastases in the group who responded well to the treatment. On the other hand, the majority (*n*: 3, 60%) of the patients had more than three sites of metastases in the group who did not respond to the OIP. Those who responded to OIP, four patients had previous one cycle of other treatment, whereas all patients who did not respond to OIP had two or more cycles of previous treatments (Table 5).

Patients received OIP for a median duration of 6.5 (IQR: 3–13.25) months, and the median PFS after OIP initiation was 5 (IQR: 3–12.75) months. Only one patient reported an adverse effect, developing cellulitis at the pump insertion site. The patients in our cohort exhibited a median OS of 14 months (IQR: 7.5–19).

## Discussion

Our study aimed to assess the effect of OIP in patients with advanced NETs and refractory hormonal syndrome, including survival benefits. Our study cohort included predominantly patients with well-differentiated midgut NETs (G1 or G2), and most patients had liver metastases. After initiating OIP therapy, 72% of patients in our cohort reported an SR in terms of the frequency of diarrhea and/or cutaneous flushing. Adverse effects were mild and included only one case of local cellulitis at the site of the infusion system entry. Fifteen (83%) patients developed symptoms of refractory hormonal syndrome despite being on long-acting SSTAs; of these, four (26.66%) patients were continued on both OIP therapy and long-acting SSTAs. The median PFS post-OIP initiation was 5 months, while the median OS from the date of initiation of OIP was 14 months (IQR: 7.5–19). To our knowledge, this is the first study evaluating the efficacy of OIP therapy in patients with a diagnosis of refractory hormonal syndrome.

The definition of resistance to medical treatment of symptoms targeting hormone excess varies between studies. A few studies defined resistance to treatment as the inability to reduce bowel movements to less than 4 per day or <20% and <30% reduction in bowel movements and flushing from baseline episodes respectively (Wolin & Benson 2019). The exact mechanism of resistance to SSTAs is still unknown. Various hypotheses propose that this phenomenon could be due to the following mechanisms (Angelousi *et al.* 2023).

(i) A consequence of heterogeneous expression of somatostatin receptors (SSTRs) in NETs. For example, several studies have confirmed that SSTAs are preferably more expressed in well-differentiated NETs compared to poorly differentiated, more aggressive NETs. The antiproliferative effects of SSTAs have been found to be more evident in patients with well-differentiated NETs with Ki67 of <10% compared to patients with NETs and Ki67 of >10% in randomized controlled trials (Reubi *et al.* 1992, Pavel *et al.* 2021).
(ii) Desensitization of SSTRs due to uncoupling from the signaling cascade. The upregulation of let-7 family, miR-148, miR-7 and miR-96 as well as downregulation of miR-3137 and miR-185 induced by SSTAs in small bowel NETs seems to interfere with the inhibition of the signaling pathway of P13K/AKT/mTOR, which is implicated in tumor development and autophagy (Bösch *et al.* 2019, Curt *et al.* 2020).
(iii) Absence of functional receptors due to mutation of the genes encoding for SSTRs. Under the influence of SSTAs, different subtypes of SSTR can form dimers at the membrane level with other members of the SSTR family. This process can alter functional properties of the receptor such as signaling, ligand binding, agonist-induced regulation and can possibly create new receptors with different functional and biological properties (Rocheville *et al.* 2000). Notably, as SSTAs can downregulate SSTR expression, long-term use can significantly affect SSTR expression (Rocheville *et al.* 2000, Angelousi *et al.* 2023). In addition, under the influence of SSTAs, dimerization between different subtypes of SSTR can also happen, which alters functional properties of receptors (Rocheville *et al.* 2000*b*). Further mechanisms such as epigenetic changes within tumor cells and mutations in *DAXX* (death-domain-associated protein) or *ATRX* (alpha thalassemia/mental retardation syndrome X-linked) have also been proposed (Angelousi *et al.* 2023).
(iv) Rarely, development of autoantibodies to long-acting SSTAs may be responsible for causing resistance to SSTAs (Kaal *et al.* 2000, Martín-Richard *et al.* 2013, Caplin *et al.* 2014).

Management of refractory hormonal syndrome in F-NETs remains a clinical challenge. While SSTAs are the mainstay therapies, they achieve complete symptom control in only 40–70% of patients (Ito *et al.* 2018, Oleinikov *et al.* 2019, Stueven *et al.* 2019, Wolin & Benson 2019). According to a recent meta-analysis, the use of octreotide and lanreotide can lead to significant symptomatic improvement in 65–72% patients (Hofland *et al.* 2019). SR can be further improved to 72–84% if the dose or frequency is increased or switched to different SSTAs (Angelousi *et al.* 2023). Moreover, as observed in our cohort of patients, symptoms attributed to well-differentiated NETs initially tend to respond well to treatment with SSTAs, but these symptoms may become resistant over time (Angelousi *et al.* 2023). The use of telotristat ethyl and interferon-alfa for symptomatic carcinoid syndrome improves symptoms in only 40% and 45–63% patients who previously received SSTAs respectively (Grozinsky-Glasberg *et al.* 2022). On the contrary, chemotherapy and everolimus use in patients with symptomatic carcinoid syndrome did not induce significant symptomatic hormonal response (Hofland *et al.* 2019). Although the use of PRRT seems promising, there is a paucity of data with regard to symptomatic control of CS (Hofland *et al.* 2019).

These data suggest that there is a need for different treatment strategies in the management of refractory hormonal syndrome in patients who do not respond to conventional therapy. One potential approach involves continuous infusion of mega-dose octreotide, which has been used safely in carcinoid crisis management without any significant complications or side effects of the therapy (Seymour & Sawh 2013). The pharmacokinetics of SSTAs support this strategy, as the shorter half-life of subcutaneous (1–2 h) and long-acting release (LAR) octreotide (23–30 days) may influence individual patient responses (La Salvia *et al.* 2023). Continuous octreotide infusion could provide more consistent symptom relief for patients with refractory hormonal syndromes.

After initiating OIP therapy, 72% of patients in our cohort reported an SR in terms of reducing the frequency of diarrhea and/or cutaneous flushing by more than 50%. This response suggests that treatment with OIP may be effective in managing symptoms that were refractory to SSTAs and other treatments. We also noted that the majority of patients who did not respond had more disease burden and had received more cycles of previous treatments, indicating the severity of the disease. The safety profile of OIP therapy in this cohort was favorable, with only one reported case of cellulitis at the pump site; none of the patients had any severe debilitating steatorrhea or gastrointestinal side effects. This outcome is consistent with the literature suggesting that adverse effects of higher-than-average approved dose SSTA therapy are typically manageable and primarily related to gastrointestinal symptoms such as abdominal bloating and discomfort (Koumarianou *et al.* 2022). When comparing the effects between two groups, we found that patients with metastatic VIPoma had generally higher starting doses of OIP compared to patients with carcinoid syndrome. This could be attributable to the small sample size or aggressive tumor behavior in patients with VIPoma. In addition, all patients with VIPoma responded well to OIP compared to ten out of 15 (66.66%) patients with carcinoid syndrome. This could imply that it may be beneficial for the patients to be started on higher doses of OIP to avoid delays in symptomatic improvement.

Evidence on the use of subcutaneous octreotide infusion can be retrieved from old data on the treatment of acromegaly before the discovery of long-acting formulations. In most studies, patients reported subjective improvement in symptoms related to acromegaly, and levels of growth hormone and insulin-like growth factor-1 (IGF-1) were found to be suppressed. Apart from mild and transient gastrointestinal upset, patients tolerated octreotide infusion therapy well (James *et al.* 1989, Tauber *et al.* 1989, Tauber *et al.* 1995). Earlier data also highlight that continuous octreotide infusion has not been shown to be associated with an increased risk of gallstone formation compared to intermittent subcutaneous octreotide therapy (Tauber *et al.* 1995). Moreover, there are reports of using subcutaneous octreotide infusion in patients with congenital hyperinsulinism and nesidioblastosis with no significant side effects or complications (Yorifuji *et al.* 2013, Kato *et al.* 2021).

There were multiple major limitations in our study. First, the sample size was very small and therefore the results cannot be generalized to all NET patients with refractory hormonal syndrome. Second, it was a retrospective case series, and the results are therefore susceptible to bias. Third, there were missing data on biochemical parameters such as urinary 5-HIAA levels and serum VIP levels before and after initiating OIP therapy.

## Conclusion

Refractory hormonal symptoms are not uncommon in NET patient populations and this is an area of unmet clinical need. Although treatment with OIP therapy might not have a significant effect in disease control, it appears to be safe and effective in controlling the severity of refractory hormonal syndrome in patients with metastatic NETs. Randomized controlled trials are required to confirm the significance of our findings.

## Declaration of interest

The authors declare that there is no conflict of interest that could be perceived as prejudicing the impartiality of the work reported.

## Funding

This work did not receive any specific grant from any funding agency in the public, commercial or not-for-profit sector.

## Author contribution statement

KMS contributed to the original draft writing, reviewing, editing and interpretation of data. EA was involved in original draft writing, review, editing and supervision. DM contributed to writing, reviewing and editing. AM was responsible for data analysis, writing of the results section, reviewing and editing. GP and AS contributed to data collection and participated in writing, reviewing and editing. AH and MC contributed to writing, reviewing and editing. CT was involved in conceptualization, writing, reviewing and editing, and provided supervision.
